# Suppression of mI_CAT_ in Mouse Small Intestinal Myocytes by General Anaesthetic Ketamine and its Recovery by TRPC4 Agonist (-)-englerin A

**DOI:** 10.3389/fphar.2020.594882

**Published:** 2020-12-18

**Authors:** Mariia I. Melnyk, Dariia O. Dryn, Lina T. Al Kury, Dmytro O. Dziuba, Alexander V. Zholos

**Affiliations:** ^1^ A.A. Bogomoletz Institute of Physiology, National Academy of Sciences of Ukraine, Kyiv, Ukraine; ^2^ Institute of Pharmacology and Toxicology, National Academy of Medical Science of Ukraine, Kyiv, Ukraine; ^3^ ESC “Institute of Biology and Medicine”, Taras Shevchenko National University of Kyiv, Kyiv, Ukraine; ^4^ Department of Health Sciences, Zayed University, Abu Dhabi, United Arab Emirates; ^5^ Shupyk National Medical Academy of Postgraduate Education, Kyiv, Ukraine

**Keywords:** ketamine, (-)-englerin A, gastrointestinal tract, G-proteins, postoperative ileus, transient receptor potential canonical subfamily member 4 channel

## Abstract

A better understanding of the negative impact of general anesthetics on gastrointestinal motility requires thorough knowledge of their molecular targets. In this respect the muscarinic cationic current (mI_CAT_ carried mainly via TRPC4 channels) that initiates cholinergic excitation-contraction coupling in the gut is of special interest. Here we aimed to characterize the effects of one of the most commonly used “dissociative anesthetics”, ketamine, on mI_CAT_. Patch-clamp and tensiometry techniques were used to investigate the mechanisms of the inhibitory effects of ketamine on mI_CAT_ in single mouse ileal myocytes, as well as on intestinal motility. Ketamine (100 µM) strongly inhibited both carbachol- and GTPγS-induced mI_CAT_. The inhibition was slow (time constant of about 1 min) and practically irreversible. It was associated with altered voltage dependence and kinetics of mI_CAT_. In functional tests, ketamine suppressed both spontaneous and carbachol-induced contractions of small intestine. Importantly, inhibited by ketamine mI_CAT_ could be restored by direct TRPC4 agonist (-)-englerin A. We identified mI_CAT_ as a novel target for ketamine. Signal transduction leading to TRPC4 channel opening is disrupted by ketamine mainly downstream of muscarinic receptor activation, but does not involve TRPC4 *per se*. Direct TRPC4 agonists may be used for the correction of gastrointestinal disorders provoked by general anesthesia.

**GRAPHICAL ABSTRACT:**
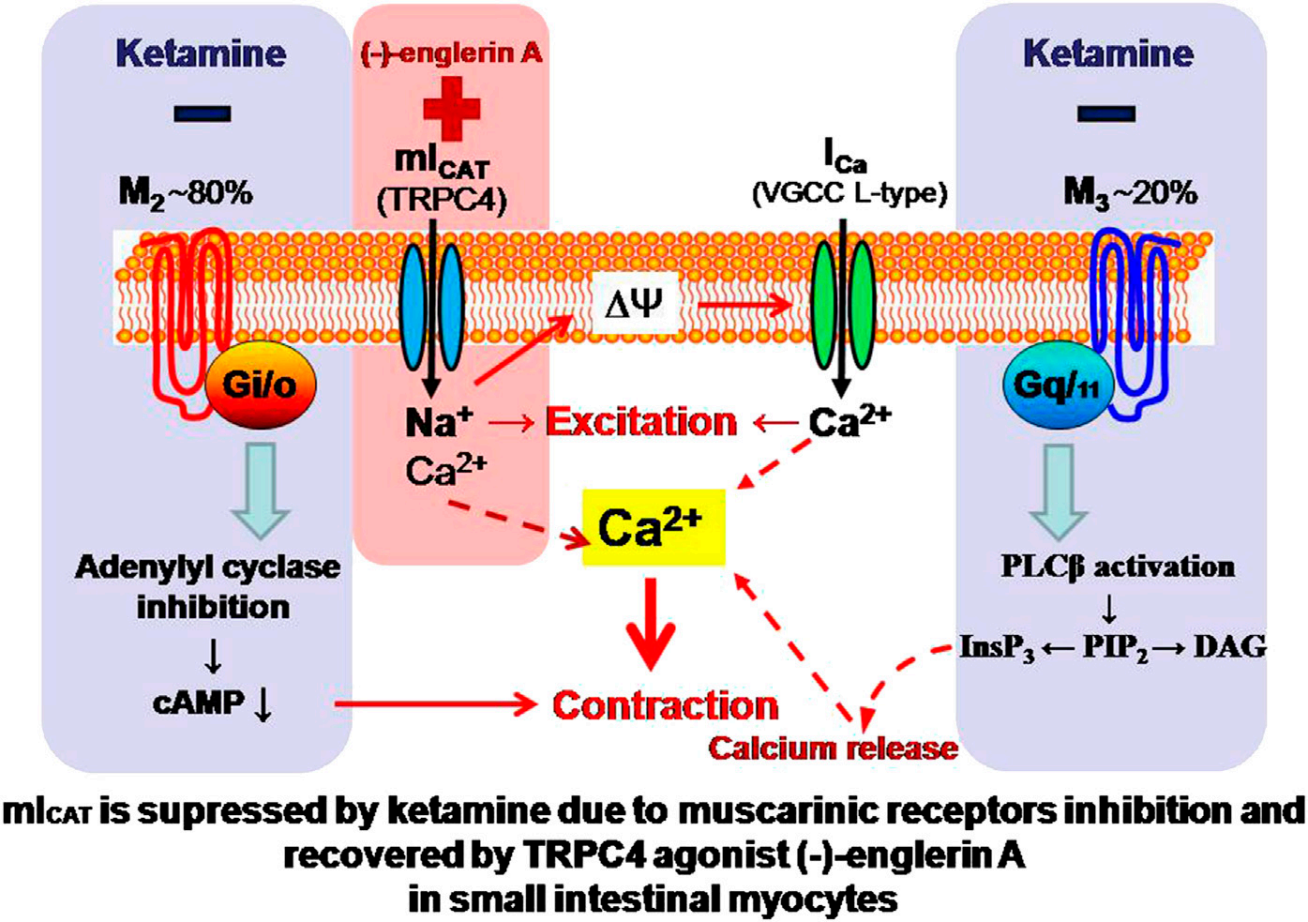


## Introduction

Ketamine (IUPAC Name: 2-(2-Chlorophenyl)-2-(methylamino)-cyclohexan-1-one), first synthesized in 1962 and included on the WHO Essential Medicines List since 1985, is commonly used as an anaesthetic and analgesic drug both for human and veterinary applications worldwide. Its anesthetic properties were first described in 1965 (Domino et al., 1965). Moreover, ketamine is now also increasingly recognized by WHO for its anti-depressant and anti-seizure benefits. The anesthetic action of ketamine is believed to mainly result from its ability to inhibit the NMDA receptor for excitatory amino acids as was initially characterised in mouse hippocampal neurons (MacDonald et al., 1987). Since then a number of other molecular targets of ketamine have been identified, including neuronal hyperpolarization-activated cyclic nucleotide channels, δ- and µ-opioid receptors, cholinergic receptors, GABA, AMPA and metabotropic glutamate receptors, the NO-cGMP signaling system, voltage-gated Na^+^ and L-type Ca^2+^-channels, and large-conductance BK_Ca_ channels (Sleigh et al., 2014; Zanos et al., 2018).

Such multiple-targeting action of ketamine can enhance its anaesthetic and analgesic properties but can also cause certain undesirable side effects. In addition, in connection with the existence of multiple molecular targets, other uses of ketamine are also being proposed owning to its anti-inflammatory and anti-depressant properties, as well as bronchodilatory effects (Duman et al., 2012; Kurdi et al., 2014; Niesters et al., 2014; Iadarola et al., 2015; Li and Vlisides, 2016).

In this study, based on our previous extensive investigation of one of such putative targets of ketamine, namely muscarinic receptor (mAChR) cation channels in the gastrointestinal (GI) tract, which initiate cholinergic-excitation contraction coupling in the gut (Zholos and Bolton, 1997; Bolton et al., 1999; Zholos et al., 2004; Tsvilovskyy et al., 2009), we aimed to test such effects of the drug on the underlying depolarizing inward current termed mI_CAT_ that is mainly carried by TRPC4 receptor-operated cation channels (Tsvilovskyy et al., 2009). In this previous study involving TRPC4 and/or TRPC6 knock-out mice, the functional impact of mI_CAT_ on carbachol (CCh)-induced depolarisation, neurogenic ileal contractions and small intestinal transit was demonstrated (Tsvilovskyy et al., 2009).

Our interest has been prompted by the still unresolved problem of one of the GI motility disorders—postoperative ileus (POI), which imposes a significant economic burden on the healthcare system (Iyer et al., 2009) and for which there is no effective pharmacological treatment (Lubawski and Saclarides, 2008; Harnsberger et al., 2019).

Thus, the aim of the present study was to systematically assess the effects of ketamine on mI_CAT_ in isolated mouse ileal myocytes. We found that ketamine at clinically relevant concentrations strongly suppressed this current. While inhibition of mAChRs is apparently not involved, downstream signaling cascades, most likely at the level of G-proteins and/or beyond, appear to be the main targets. Most interestingly, application of TRPC4 direct agonist (-)-englerin (EA) can fully overcome ketamine-induced inhibition of mI_CAT_. These results have been communicated in an abstract form at the Physiology-2019 main meeting (Dryn et al., 2019).

## Material and Methods

### Animals

Animal studies were carried out in accordance with the recommendations of the EU directive 2010/63. They also confirm to the guidelines of the UK Animals (Scientific Procedures) Act 1986 and are reported in compliance with the ARRIVE guidelines.

All experiments were performed on BALb/c two month-old (25–30 g) wild type male mice housed under normal environmental conditions at 21°C, 12 h light-dark cycle, with free access to water and standard rodent chow. The mice were humanely euthanized by cervical dislocation. The total number of mice used was approximately 40.

### Cells Isolation

The ileum longitudinal smooth muscle (SM) layer was rapidly removed by carefully peeling the SM in the longitudinal direction and placed into a normal physiological salt solution (PSS) containing (in mM): NaCl 120, glucose 12, HEPES 10, KCl 6, CaCl_2_ 2.5, MgCl_2_ 1.2, pH 7.4 (adjusted with NaOH). SM cells were isolated by enzymatic digestion using (in mg/ml) 1 collagenase type 1A, 1 soybean trypsin inhibitor II-S, 1.5 bovine serum albumin in divalent cation-free PSS following tissue incubated for 18 min at 36.5°C, as described in more detail elsewhere (Dryn et al., 2018).

### Electrophysiological Studies

Membrane currents were recorded in the whole-cell configuration of the patch-clamp techniques at room temperature (22–25°C) using an Axopatch 200B voltage-clamp amplifier (Molecular Devices, San Jose, CA, United States). Voltage-clamp pulses were generated and data were captured using a Digidata 1322A interfaced to a computer running the pClamp 8 program (Molecular Devices, San Jose, CA, United States). Patch pipettes, made from borosilicate glass (1.5 mm OD, 0.86 mm ID, Sutter Instrument Corp., Novato, CA, United States) using a P-97 Flaming/Brown micropipette puller (Sutter Instrument Corp., Novato, CA, United States), had a resistance of 2.5–4 MΩ when filled with an intracellular solution.

Whole-cell data were filtered at 2 kHz and sampled at 10 kHz for analysis. Series resistance was compensated for by ∼40%. Holding potential was −40 mV. The steady-state current-voltage relationships of mI_CAT_ were measured by applying either 1.2 s duration voltage steps to test potentials ranging from −120 to 80 mV with a 10 mV increment at 5 s interval or by slow voltage ramps from 80 to −120 mV lasting 6 s, which were applied every 30 s.

Single-channel activity was recorded in the outside-out configuration of the patch-clamp techniques. Membrane patches were formed by slowly withdrawing the pipette from the cell after whole-cell mI_CAT_ was induced by 50 µM CCh. These currents were filtered at 1 kHz and digitized at 10 kHz. For the illustration purposes they were decimated with factor of 10. Mean patch current was calculated as current integral after baseline current correction to bring any background current to zero.

Before current recordings cells were kept in PSS, while for recordings of mI_CAT_ the bath solution was replaced with a Cs^+^-containing solution (in mM): CsCl 120, glucose 12, HEPES 10, pH 7.4 (adjusted with CsOH). The pipette solution contained (in mM): CsCl 80, MgATP 1, creatine 5, glucose 5, BAPTA 10, HEPES 10, CaCl_2_ 4.6, pH 7.4 (adjusted with CsOH). Such symmetrical Cs^+^-containing solutions help to isolate and maximize mI_CAT_ while avoiding its intracellular Ca^2+^-dependent modulation (Gordienko and Zholos, 2004) as the intracellular free Ca^2+^ concentration ([Ca^2+^]_i_) was “clamped” at 100 nM by the BAPTA/Ca^2+^ buffer. mI_CAT_ was activated either by 50 µM CCh or, to bypass M2/M3-receptors, by an infusion of 200 μM GTPγS added to the pipette solution. BPS-8 solution exchange system (ALA Scientific Instruments, Inc., New York, NY, United States) was used for solution application. Complete exchange of the external solution was estimated to take about 1 s.

### Intact Tissue Isometric Force Measurement

The entire thickness ileum segments (2 cm long) of the distal section of the small intestine were dissected, mounted in a 2-ml organ bath and continuously superfused using a peristaltic pump with a modified Krebs-bicarbonate buffer solution (in mM): NaCl 133, KCl 4.7, NaHCO_3_ 16.3, NaH_2_PO_4_ 1.38, CaCl_2_ 2.5, MgCl_2_ 1.2, HEPES 10, d-glucose 7.8, pH adjusted to 7.4 with NaOH. Temperature was maintained at 37°C. After a 40 min equilibration period at the resting tension of 0.5 g the tissue isometric contractions were recorded by an external force displacement transducer (AE 801, SensoNor A/S, Norten, Norway) connected to an AD converter Lab-Trax 4/16 (World Precision Instruments, Inc., Sarasota, FL, United States). The data were acquired at 10 kHz sampling frequency using DataTrax2 software (World Precision Instruments, Inc., Sarasota, FL, United States).

### Materials

All reagents were obtained from Sigma Chemical (St. Louis, MO, United States), except the ketamine purchased from Farmak Joint-Stock Co. (Kyiv, Ukraine).

### Measurements and Statistical Analysis

Different experimental protocols (e.g. treatments with CCh, GTPγS or EA) were performed on the same day to reduce the number of animals. No technical replicates (e.g. using identical protocols) were made, groups for comparisons were of equal size and no outliers were excluded from data analysis. SM preparations and cells were randomly assigned to various tests and data analysis was performed independently in a semi-blinded manner whenever possible (it should be noted that the inhibitory effects of ketamine were fairly obvious to the operator even without data analysis). Group size was determined based on our previous extensive experience studying mI_CAT_, and in particular on our recent similar study of mI_CAT_ inhibition produced by isoflurane (Dryn et al., 2018). Current amplitudes were normalized by cell capacitance to account for variations in cell size. In experiments with ketamine, responses before and after drug application were compared in the same cell by calculating their ratio. This approach using matched controls would allow power of 0.89 (if *n* = 5) and power of 0.96 (if *n* = 6) to be achieved at *α* = 0.05 (the probability of rejecting the null hypothesis when it is true), if, for example, current density was reduced from −14.1 pA/pF to −7.9 pA/pF and standard deviation of the differences for paired samples was 3.2, as was the case for ketamine (100 µM) inhibition of GTPγS-induced current.

Data were analyzed and plotted using Clampfit 8 (Molecular Devices, Sunnyvale, CA, United States) and OriginPro 2020b (v. 9.7.5.184) software (OriginLab Corporation, Northampton, MA, United States). Descriptive data are given as mean (standard error of the mean) with *n* indicating the number of preparations/cells used for a particular set of measurements. For statistical comparisons we used two-sided Student's *t*-test (for two groups) or in case of the relatively small sample sizes non-parametric tests that do not require the assumption of normal distribution of experimental values as detailed on the OriginLab Homepage describing non-parametric statistics (OriginLab, 2020). In this case, two groups were compared using the Mann–Whitney test for independent samples or the Wilcoxon signed rank test for paired samples. Multiple groups were compared using Friedman ANOVA. Confidence intervals where appropriate (e.g. for quotient of two means) were calculated using GraphPad online tool (GraphPad QuickCalcs, 2020). Differences were considered significant at *p* < 0.05.

## Results

### Effects of Ketamine on Muscarinic Cation Current Evoked by Carbachol or Intracellular GTPγS

Experimental conditions used in this study allow efficient isolation of mI_CAT_ since all K^+^ currents are abolished using symmetrical Cs^+^ based solutions, while voltage-dependent Ca^2+^ current is abolished in Ca^2+^-free external solution. In addition, as already mentioned, any Ca^2+^-dependent modulation of mI_CAT_ is prevented by “clamping” intracellular free Ca^2+^ concentration at 100 nM using 10 mM BAPTA/4.6 mM Ca^2+^ mixture. As we showed previously, under these conditions currents, in response to either external carbachol or intracellular GTPγS application, show similar biophysical properties, such as double rectification around the reversal potential and U-shaped I-V curve at negative potentials, which are hallmarks of TRPC4-mediated currents (Zholos and Bolton, 1994; Zholos and Bolton, 1996; Zholos and Bolton, 1997; Zholos et al., 2004; Zholos et al., 2004). Moreover, this current is inhibited by M2-and M3-selective muscarinic receptor antagonists (Zholos and Bolton, 1997), intracellular GDPβS, pertussis toxin treatment and anti-G_αi3_/G_αo_ antibodies (Pucovsky et al., 1998; Yan et al., 2003). Moreover, mI_CAT_ is strongly suppressed in TRPC4^−/−^ mice and it is completely abolished in TRPC4^−/−^/TRPC6^−/−^ mice (Tsvilovskyy et al., 2009).

Two different approaches for the activation of TRPC4 channels mediating mI_CAT_ have been used to identify the molecular targets involved in ketamine-dependent inhibition of the current. These channels are known to be activated in synergy by M2 and M3 mAChRs, which are differentially coupled to G_i/o_ and G_q/11_ proteins, respectively (Zholos and Bolton, 1997). To differentiate between mAChR/G-protein gated and solely G-protein activated mI_CAT_ we employed two different strategies for mI_CAT_ activation. Following the first one, mI_CAT_ was activated by CCh (at sub-maximal concentration of 50 μM) that initiated signal transduction at the mAChR level. Following the second one, infusion of GTPγS (200 μM) into isolated mouse ileal myocytes via a patch-pipette was used. In the latter case, mAChRs are completely bypassed as GTPγS directly activates all available trimeric G-proteins slowly and irreversibly.

**FIGURE 1:**
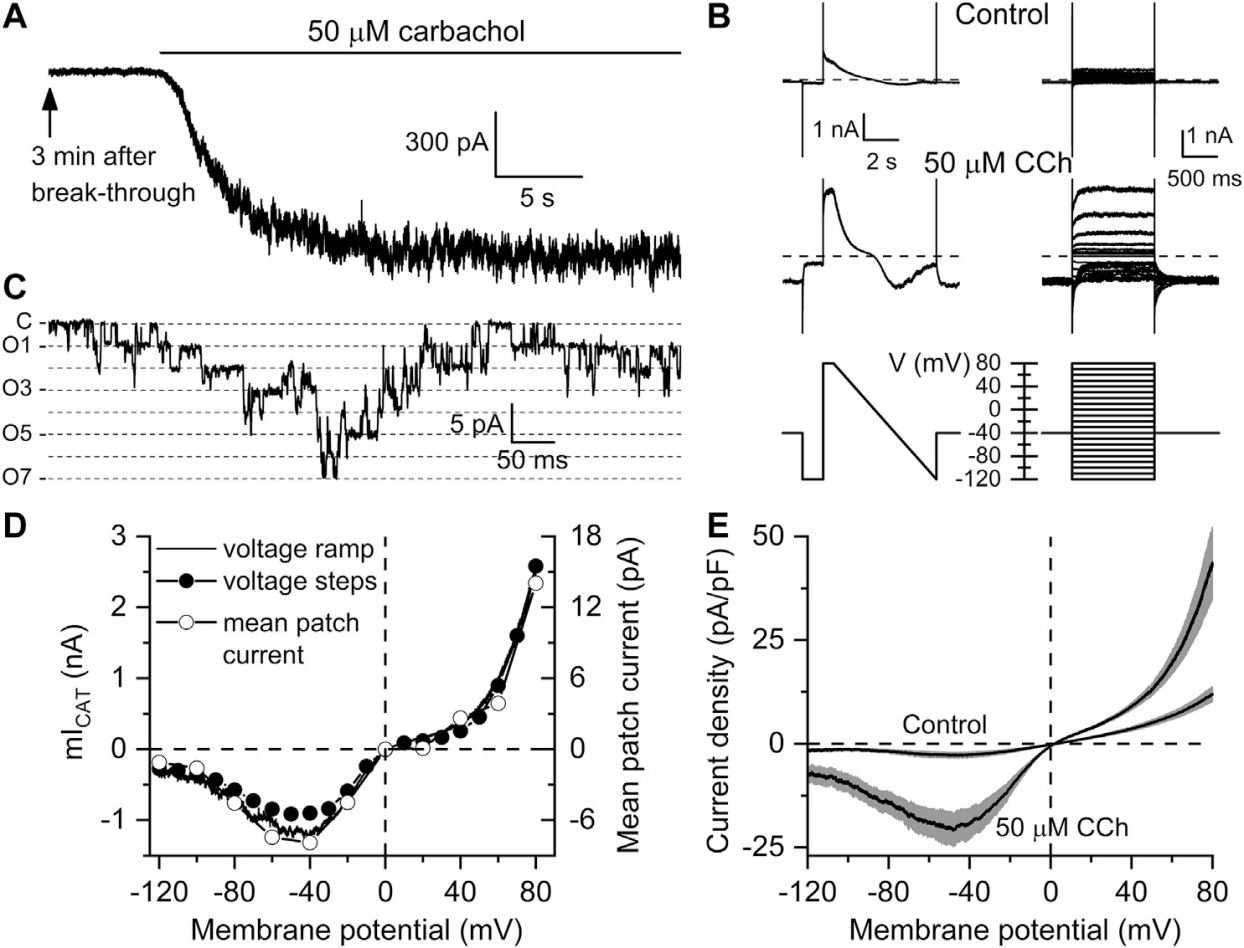
Kinetics and voltage dependence of carbachol-induced mI_CAT_ in mouse ileal myocytes. **(A)** Typical recording showing that 3 min after break-through (to allow complete equilibration of the cytosol with the pipette solution containing 125 mM Cs^+^ that abolished any K^+^ currents) carbachol applied at sub-maximal concentration of 50 µM induced a large inward current at the holding potential of −50 mV. **(B)** Current kinetics and voltage dependence were assessed in the same cell shortly before carbachol application (top, control traces) and after mI_CAT_ reached peak amplitude (middle traces) using two different protocols shown in bottom panels, either by applying slow (6 s) voltage ramp from 80 to −120 mV (bottom left panel) or a series of 1.2 duration voltage steps applied from a holding potential of −40 mV to different test potentials ranging from −120 to 80 mV with a 10 mV increment at 5 s intervals (bottom right panel). **(C)** in the presence of the muscarinic agonist, outside-out patch excision from the same cell revealed activity of multiple cation channels (C—closed state level, O1-O7—multiple openings as shown by dashed lines) of 60 pS single-channel conductance that mainly mediate mI_CAT_ (Dresviannikov et al., 2006; Tsvilovskyy et al., 2009). **(D)** I–V relationships of mI_CAT_ measured in the same cell with voltage ramp, at the end of each voltage step with voltage steps, as well as mean patch currents recorded at different test potentials, as indicated. **(E)** Mean I–V curves measured in Ca^2+^, Mg^2+^-free, Cs^+^ containing external solution 3 min after break-through in control and at the peak response to 50 µM carbachol (*n* = 8 in each case). Grey bands show S.E.M. values. In each cell, current amplitude was normalized by cell capacitance to calculate current density expressed in pA/pF. Mean cell capacitance was 33.9 ± 2.2 pF (*n* = 8).

Figure 1 illustrates measurements of carbachol-induced currents. Specifically, Figure 1A shows typical example of the time course of mIcat activation by 50 µM carbachol, while I-V curves measured in the same cell using voltage steps or voltage ramp protocols before and after carbachol application are shown in Figure 1B. In the same cell, activity of the major 60 pS channel mediating this current (Dresviannikov et al., 2006; Tsvilovskyy et al., 2009) was recorded in an outside-out patch excised from the same cell (Figure 1C). This channel was rigorously identified as TRPC4 in TRPC4^−/−^ mice (Tsvilovskyy et al., 2009). TRPC4 mediates >80% of the whole-cell mIcat as was found in that study. The I–V curves for the whole-cell currents obtained with two different voltage protocols together with the I–V curve for the mean patch current are shown in Figure 1D, as well as I–V curves measured in control, that is 3 min after break-through and just before carbachol application, and I–V curves of currents induced in the same cells (*n* = 8) after carbachol application (Figure 1E).

**FIGURE 2:**
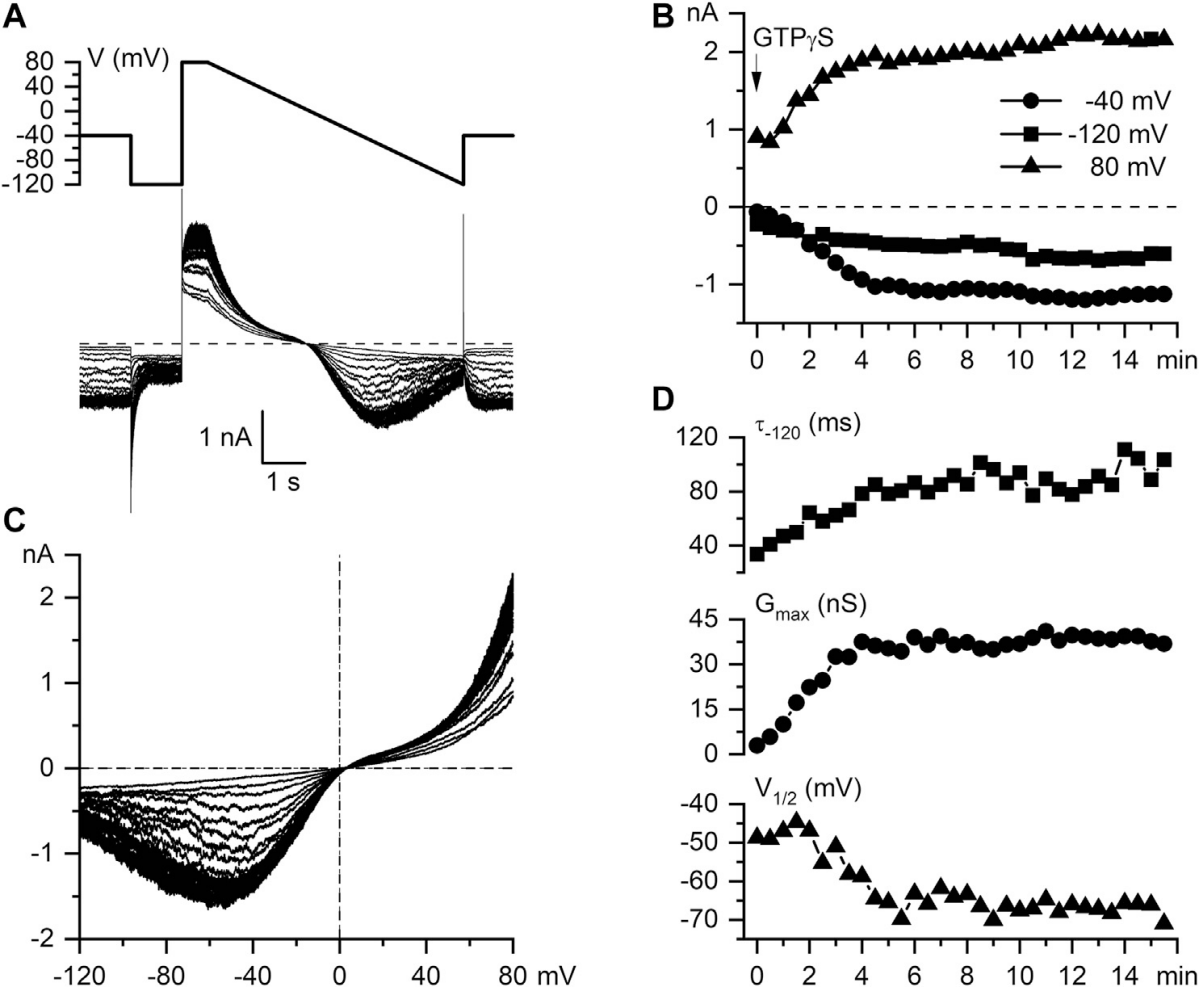
Biophysical properties of intracellular GTPγS-induced mI_CAT_ in mouse ileal myocytes. **(A)** Voltage protocol and superimposed current traces recorded in a mouse ileal myocyte at 30 s interval following break through with a patch pipette containing 200 µM GTPγS. **(B)** Time course of current amplitudes measured at −40 mV (holding potential), −120 mV, and 80 mV, as indicated. **(C)** Superimposed mI_CAT_ I–V curves measured by slow voltage ramps. **(D)** Top panel, time constant of GTPγS-induced mI_CAT_ deactivation during voltage step from -40 to -120 mV; middle and bottom panels, best-fit parameters of the Boltzmann equation fit of the cation conductance curves: the maximal conductance (G_max_) and potential of its half-maximal activation (V_1/2_), respectively.

Having rigorously established appropriate experimental conditions for carbachol-induced mI_CAT_ isolation and analysis we further examined intracellular GTPγS-induced currents in mouse ileal myocytes and found that these were highly similar to CCh-induced currents with regard to their overall voltage dependence (e.g. double rectification around 0 mV, U-shaped curve at negative potentials) and voltage-dependent relaxations kinetics. Thus, Figure 2A illustrates superimposed current traces recorded at 30 s interval following break through with of GTPγS (200 μM) added to the pipette solution using the same voltage ramp protocol as in Figure 1B. This maneuver produced a slowly rising mI_CAT_ that reached its maximal amplitude typically during 5–7 min from the onset of cell perfusion as shown in Figure 2B. The slow time course of current activation allowed us to repeatedly evaluate biophysical properties (voltage dependence, kinetics) of GTPγS-induced mI_CAT_ by applying voltage steps to three different test potentials −40 (also holding potential), −120 and 80 mV, as well as slow voltage ramps from 80 to −120 mV at 30 s interval (Figure 2A, top panel). The wide range of test potentials was required for the assessment of voltage dependence of mI_CAT_ and its possible alteration by ketamine, while current amplitude at the holding potential of −40 mV, which is close to the normal resting potential of these cells, was the most relevant functionally. Accordingly, Figure 2B illustrates typical time course of current development at the three test potentials (e.g. mean current amplitudes during the last 100 ms segment at each test potential plotted vs. time), while Figure 2C shows superimposed current-voltage (I–V) relationships measured in the whole range of potentials by the slow voltage ramp. These were doubly-rectifying around the reversal potential (E_REV_ was close to 0 mV in symmetrical Cs^+^-containing solutions used) and U-shaped at negative potentials, which is typical for mI_CAT_ (Figure 2C).

**FIGURE 3:**
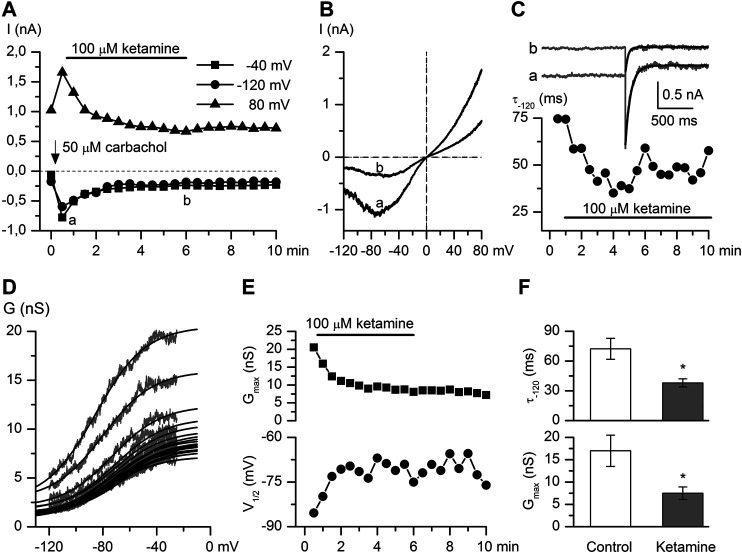
Ketamine strongly inhibits CCh-induced mI_CAT_. **(A)** Time course of mI_CAT_ evoked by CCh (50 μM) and inhibited by ketamine (100 μM) application at three different test potentials, as indicated. **(B)** Representative steady-state I–V relationships of mI_CAT_ at maximum response to CCh and during maximal current inhibition by ketamine (i.e. at time points a and b indicated in panel A). **(C)** mI_CAT_ voltage-dependent deactivation kinetics during voltage step from −40 to −120 mV fitted by single exponential function (superimposed smooth black lines) at the same time points marked a and b in panel **A**. **(D)** Cation conductance activation curves were fitted by the Boltzmann equation shown by the superimposed smooth black lines. **(E)** Ketamine not only markedly reduced the maximal conductance (G_max_) (top panel), but also shifted the potential of half-maximal activation (V_1/2_) positively (bottom panel). **(F)** Mean deactivation time constant of mI_CAT_ (top) and Gmax (bottom) in control and at steady-state inhibition by ketamine.

The rate of current deactivation during voltage steps from −40 to −120 mV is an indication of mean channel open dwell time at −40 mV (Figure 2D, top panel). mI_CAT_ deactivation kinetics were clearly altered along with the accumulation of G-proteins activated by GTPγS in the cell, as shown in Figure 2D, top panel. From the steady-state I–V relationships cation conductance activation curves were constructed (by dividing current amplitude by electrochemical driving force at each potential), that could be fitted by a Boltzmann-type equation (see Figure 3D for an example of such analysis). Best-fit parameters, namely maximal conductance (Gmax) and the potential of half-maximal activation (V_1/2_) are plotted in Figure 2D, middle and bottom panels, respectively. These reference data obtained in control cells show 1) prolongation of mean open dwell time and 2) some negative shift of the activation curve of muscarinic cation conductance along with gradual activation of G-proteins by GTPγS. The data are representative of 14 measurements in different cells summarised elsewhere (Dryn et al., 2016). We illustrate these phenomena here in control in order to contrast them later with similar measurements in the presence of ketamine.

CCh is a stable analogue of acetylcholine, the primary neurotransmitter of the parasympathetic nervous system, which binds to mAChRs and enhances TRPC4 channel activity through G-protein mediated signaling pathways including PLC activation (Zholos et al., 2004), depletion of PIP_2_ and direct channel gating by the activated Gi/o α-subunits (Yan et al., 2003; Jeon et al., 2008; Otsuguro et al., 2008; Jeon et al., 2012; Kim et al., 2012; Dryn et al., 2016). CCh was applied at its sub-maximal concentration of 50 μM (Zholos and Bolton, 1997; Yan et al., 2003). CCh-induced current amplitudes measured at −40, −120, 80 mV potentials were markedly decreased in a time-dependent manner after ketamine (100 μM) application. It should be noted that the effect developed slowly and there was no or very little recovery of the response after the ketamine wash-out (Figure 3A). CCh-activated mI_CAT_ with a mean peak amplitude of −556.5 ± 92.9 pA at the holding potential of −40 mV was reduced to −199.9 ± 22.6 pA (*n* = 8; *p* = 0.002) (see Figure 3B for an example). In parallel, voltage-dependent relaxation kinetics during voltage step from −40 to −120 mV was accelerated indicating considerable shortening of the mean open dwell time (Figure 3C, mean time constant was reduced from 72.3 ± 10.6 ms to 38.0 ± 4.1 ms, control vs*.* ketamine; Wilcoxon signed rank test for paired samples, *n* = 6, *p* < 0.05), while activation curve progressively shifted towards less negative potentials (Figure 3D) along with declining Gmax as best-fit Boltzmann equation parameters plotted in Figure 3E show. On average, Gmax was reduced from 17.0 ± 3.5 nS to 7.5 ± 1.4 nS, V_1/2_ shifted from −70.4 ± 7.2 mV to −61.7 ± 7.4 mV, or by 8.7 ± 2.0 mV positively (control vs*.* ketamine, *n* = 6, Wilcoxon signed rank test for paired samples, *p* < 0.05).

These changes in biophysical properties of mI_CAT_ are similar in magnitude, but opposite in direction compared to those that occur during gradual G-protein activation (compare to Figure 2D). Taken together with the slow development of current inhibition one can conclude that ketamine acts as a modifier of channel gating, rather than a direct blocker of TRPC4 channel pore, and that reduced G-protein activity is somehow involved.

**FIGURE 4:**
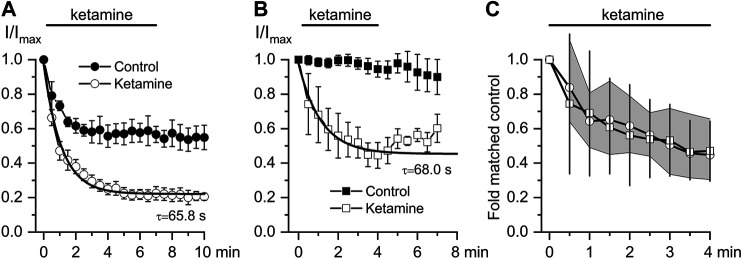
Comparison of ketamine action on mI_CAT_ evoked by CCh or GTPγS. **(A**, **B)** Time courses of average normalized 50 μM CCh-induced mI_CAT_ (a, *n* = 8) and 200 μM GTPγS-activated mI_CAT_ (b, *n* = 6) in control (closed circles), during ketamine (100 μM) application (open circles) and after its wash-out. Superimposed smooth lines show single exponential fit of the data points with the time constant values indicated (Mann–Whitney Test: *p* < 0.05 for both). **(C)** Quantification of the degree and kinetics of mI_CAT_ inhibition by ketamine showing similar effects of ketamine on both CCh- (circles) and GTPγS- (squares) induced currents. 95% CI is shown by the grey area for CCh- and by vertical lines for GTPγS-activated mI_CAT_.

Thus, we next attempted to delineate the relative involvement of mAChRs *vs.* G-proteins by comparing the inhibitory effect of ketamine for CCh- vs*.* GTPγS-induced currents. As already mentioned, GTPγs interacts directly with G-proteins avoiding mAChRs resulting in very little current desensitization while desensitisation with CCh was rather substantial (compare current time courses in control shown by closed symbols in Figures 4A,B and also note that traces shown in Figure 3A illustrate one example corresponding to the averaged data shown by the open circles in Figure 4A). The normalized maximum CCh-induced current (I_max_) at −40 mV was −15.1 ± 3.3 pA/pF in control, while at steady-state ketamine-induced inhibition its amplitude (I_inh_) was −5.5 ± 0.9 pA/pF (*n* = 8; *p* = 0.014) (Figure 4A). At the same concentration of ketamine mI_CAT_ induced by intracellular GTPγS was reduced from I_max_ −349.6 ± 19.9 pA in control to the steady-state current level of −197.1 ± 41.7 pA (*n* = 6; *p* = 0.008). Corresponding current densities were −14.1 ± 1.2 pA/pF in control and −7.9 ± 1.3 pA/pF after ketamine application (*p* = 0.006). Both the degree and kinetics of current inhibition by ketamine were practically identical in both cases (Figure 4C). Such kinetics of current inhibition is slow enough to exclude any direct blockage of the channel pore by the drug.

Preliminary dose-response studies were performed within the limits of ketamine cytotoxicity, which occurs at concentrations higher than 1 mM (Baker et al., 2016). Ketamine showed dose-dependent inhibition of CCh-induced mI_CAT_: the degree of current inhibition recorded at the holding potential of −40 mV at 1 mM was by 75.7 ± 3.7%, at 100 μM – 62.4 ± 2.6%, at 10 μM – 55.7 ± 3.6%, and at 1 μM – 37.2 ± 10.3% (*n* = 5–8 for each concentration).

**FIGURE 5:**
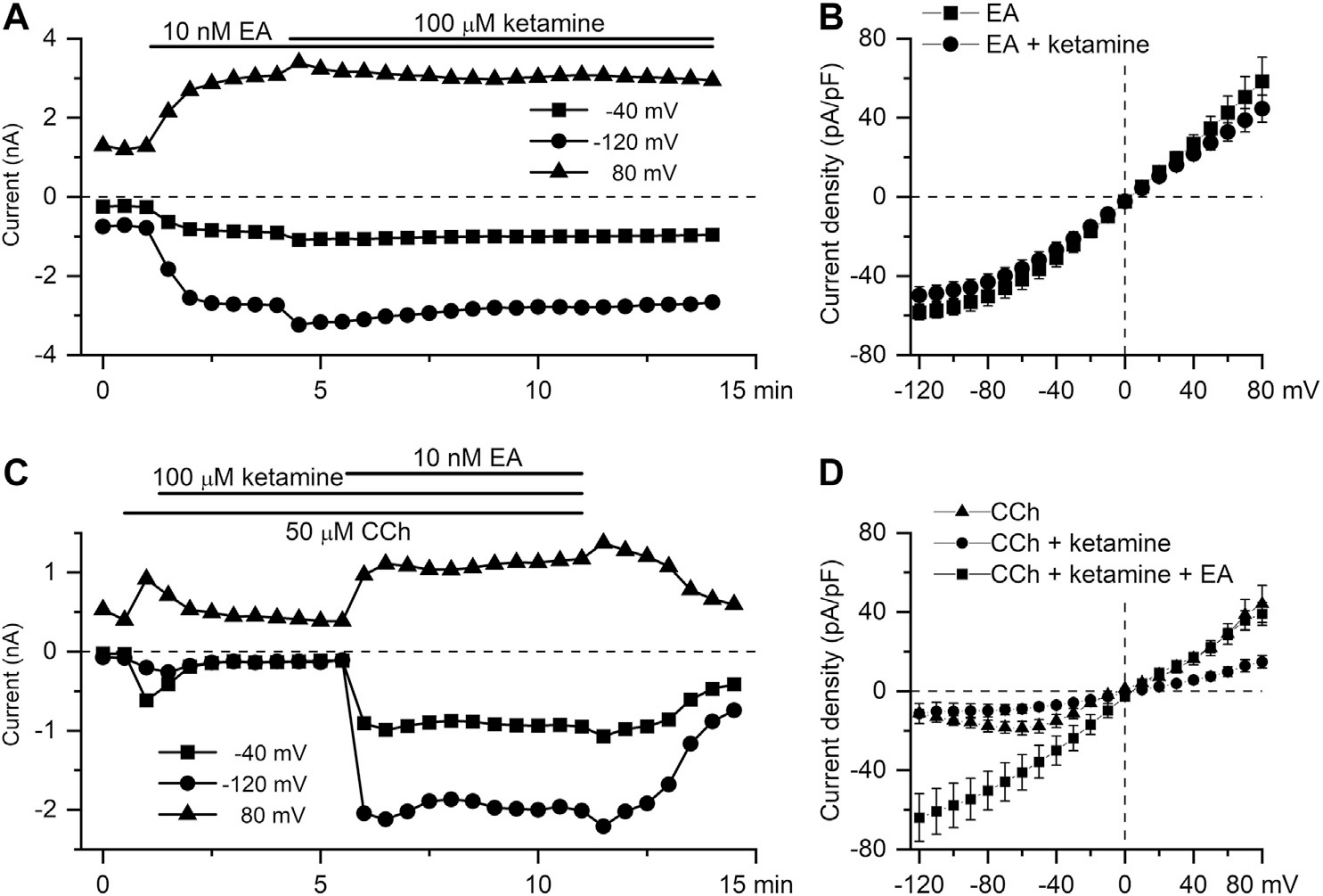
EA-induced TRPC4 current is insensitive to ketamine. **(A)** Time course of TRPC4 current evoked by EA (10 nM) at three different test potentials, as indicated, showing the lack of the inhibitory action of ketamine applied at 100 μM. **(B)** Mean steady-state I–V relationships of EA-induced current (10 nM) in control (squares, *n* = 5) and in the presence of ketamine (100 μM) (circles, *n* = 5). **(C)** EA applied at 10 nM recovers mI_CAT_ activated by the application of CCh (50 µM) and then suppressed by ketamine (100 µM). **(D)** Mean steady-state I–V relationships of CCh-induced current (50 μM) in control, in the presence of ketamine (100 μM) and after application of EA, as indicated (*n* = 5).

EA has recently been identified as a highly selective and potent direct activator of TRPC4 channels (Akbulut et al., 2015; Minard et al., 2018). It was used to further clarify the mechanism of ketamine action. Thus, application of ketamine at the peak response to EA (10 nM) did not cause any significant reduction of TRPC4 current amplitude in the whole range of membrane potentials tested (Figures 5A,B). Thus, EA-induced current density at −40 mV was −30.8 ± 4.6 pA/pF in control declining non-significantly to −26.9 ± 3.9 pA/pF after ketamine application (Figure 5B, *n* = 5; *p* = 0.536). Moreover, Figure 5C shows that mI_CAT_ activated by CCh (average amplitude of −585.2 ± 90.1 pA) and then inhibited by ketamine (mean amplitude of −287.4 ± 46.6 pA, decrease by 53%, *n* = 5; *p* = 0.019), could be efficiently restored by EA (10 nM) application. In fact, EA in the presence of ketamine increased mI_CAT_ significantly by 180% to −1,352.6 ± 349.7 pA (*n* = 5; *p* = 0.017). Current densities at −40 mV were −15.2 ± 2.6 pA/pF, −7.3 ± 1.4 pA/pF and −37.4 ± 8.1 pA/pF, in control, in the presence of ketamine and in the presence of ketamine and englerin A, respectively (Figure 5D, *n* = 5).

### Effects of Ketamine on Spontaneous and Carbachol-Induced Contractions of Small Intestine

**FIGURE 6:**
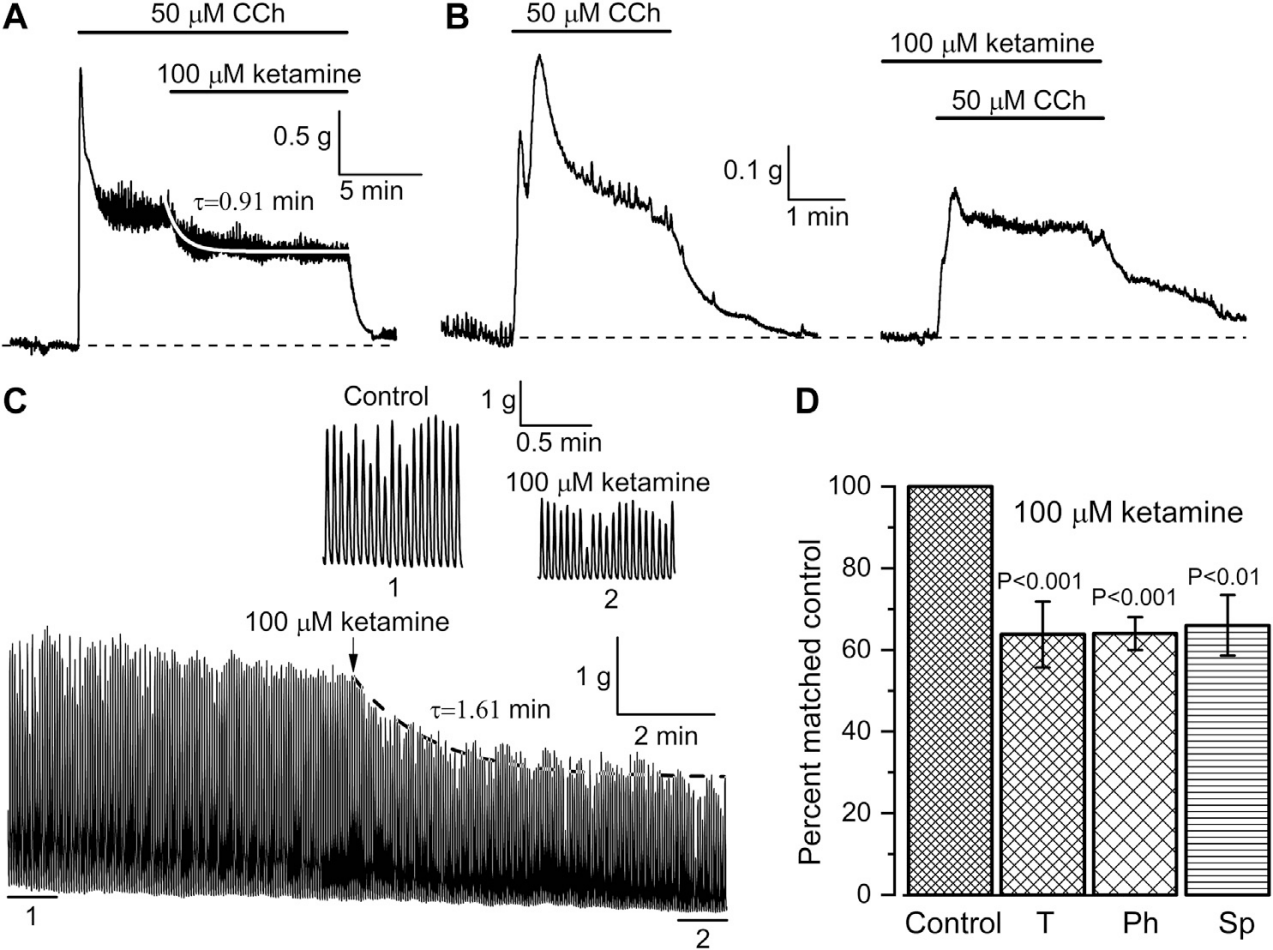
Ketamine inhibits both CCh-induced and spontaneous mouse ileum contractions. **(A**, **B)** Representative traces illustrating isometric contractions of ileum segments in response to CCh (50 μM) followed by ketamine application (100 μM) in the continuous presence of CCh (A, *n* = 9), or, alternatively, CCh application following pretreatment with ketamine (B, *n* = 10). Ketamine inhibited tonic component of the CCh-induced contraction with time constant of 0.91 ± 0.09 min as shown by the smooth white superimposed line in panel (A). **(C)** Ketamine (100 μM) also inhibited spontaneous rhythmic contractions of the ileum with time constant of 1.61 min; 1 min duration segments marked 1 (control) and (2) at the steady-state inhibition by ketamine are shown on an expanded time scale. **(D)** Summary of the inhibitory effects of ketamine on tonic (T, *n* = 9) and phasic (Ph, *n* = 10) components of CCh-induced contractions of the ileum and on its spontaneous (Sp, *n* = 7) contractions.

Further functional tests of the ketamine action were performed using *in vitro* intact tissue isometric force measurements. Two different protocols of CCh and ketamine applications were used and ketamine invariably caused inhibition of SM contractile activity in all muscle strips tested. In the first series of these experiments, the whole ileum segments were first pre-contracted with CCh (50 μM). When CCh-induced contractions reached a plateau the ketamine (100 μM) was applied resulting in the inhibition of the tonic component of SM contraction by 36.2 ± 8.1% (*n* = 7; *p* < 0.001) (Figures 6A,D). The inhibition developed with a time constant of 0.91 ± 0.09 min as shown by the white superimposed line in Figure 6A, which was compatible with its similarly slow effect on mI_CAT_ (Figures 4A,B). In the second series of experiments, CCh was applied in control and then after about 20 min pre-treatment with ketamine, as illustrated in Figure 6B. Inhibition of phasic component by 36.0 ± 4.0% (*n* = 10; *p* < 0.001) was evident in this case (Figure 6D), whereas tonic component was not significantly affected (Figure 6B). These results generally concur with the above described inhibition of mI_CAT_ by ketamine.

Inhibition of spontaneous SM contractions by ketamine was also evident and thus after ketamine application basal contractile activity decreased by 34.0 ± 7.4% (*n* = 7; *p* = 0.004) (e.g. Figures 6C,D). The inhibition developed with time constant of about 1.6 min (superimposed dotted line in Figure 6C). Parameters of the spontaneous oscillatory contractions shown in Figure 6C were compared in control and at their steady-state inhibition by ketamine: peak amplitude—2.96 ± 0.03 g vs*.* 1.70 ± 0.02 g; duration expressed as full width at half maximum ((FWHM)—976 ± 4 ms vs*.* 837 ± 5 ms; area under the curve (i.e. muscle work)—3.05 ± 0.03 g × s vs*.* 1.50 ± 0.02 g × s (control vs*.* ketamine, 86 tension oscillations were analysed in each case; Mann–Whitney test for independent samples, *p* < 0.05). This comparison is summarised in Figure 6D. In contrast, frequency of these spontaneous contractions remained unaltered (Figure 6C, portions of the trace marked 1 & 2 showing these on an expanded time scale).

## Discussion

Ketamine is one of the most widely-used unique dissociative anesthetics and an important component of balanced anesthesia. Of major relevance to the present study are its side effects related to GI motility. Patients with chronic ketamine abuse present with problems caused by SM relaxation in the upper GI tract (Pappachan et al., 2014). In medical literature clear cut, albeit isolated, cases can be found describing e.g. a profound paralytic ileus developing in patients treated with ketamine for severe bronchospasm (Amoroso and Best, 1989). Another more recent study showed that when ketamine-predominant, opioid minimizing perioperative pain control protocol was used the median time to return of bowel function was 3 days, as opposed to 6 days with an opioid-predominant analgesic regimen (Jacobsohn et al., 2015). Randomized controlled trials showed the incidence of ileus associated with intravenous ketamine use for postoperative analgesia in patients undergoing laparoscopic cholecystectomy (Ye et al., 2017). Impairment of GI motility by ketamine has also been shown in several animal models, e.g. in pigs and horses *in vivo* (Schnoor et al., 2005; Elfenbein et al., 2011), but not in dogs (Fass et al., 1995).

Here, we show for the first time that 1) 100 μM ketamine similarly inhibits both CCh- and GTPγS-induced mI_CAT_ (Figure 4C) suggesting that mAChRs *per se* are not the major targets of its action, at least under our experimental conditions; and 2) a novel potent agonist of TRPC4 channels, EA at 10 nM can fully recover CCh-induced mI_CAT_ inhibited by 100 μM ketamine (Figure 5). Strong suppression of mI_CAT_ under conditions when mAChR function is bypassed via direct activation of trimeric G-proteins by GTPγS strongly implies that the inhibition occurs at the level of G-proteins or related downstream pathways, such as PLC. Indeed, the latter is critically important for mI_CAT_ generation (Zholos et al., 2004). mI_CAT_ is also a strongly [Ca^2+^]_i_-dependent current (Gordienko and Zholos, 2004) and ketamine was shown to inhibit noradrenaline-induced Ca^2+^ release in mesenteric resistance arteries (Akata et al., 2001), but in our experiments we can safely exclude from consideration impairment of Ca^2+^-signalling pathways by ketamine when measuring mI_CAT_ since [Ca^2+^]_i_ was strongly buffered at 100 nM using 10 mM BAPTA. Altered voltage-dependence and kinetics of mI_CAT_ in the presence of ketamine (compare Figures 2 and 3) provide additional lines of evidence favoring inhibition of G-proteins. In this context, it should be noted that ketamine has high lipid solubility as it is a lipophilic base that can thus easily reach intracellularly located targets.

Overall, ketamine induced inhibition of mI_CAT_ is highly reminiscent of the effects of isoflurane (Dryn et al., 2018). To our knowledge, our experiments provide first indication that G-proteins can be inhibited by ketamine, but obviously much further work is required to clarify the underlying mechanism. In other studies it was shown that Gα_q_-subunit was the main target point of local anesthetics' action on M1 receptors (Hollmann et al., 2001) and in case of M3 receptors there was combined involvement of an intracellular charged site on Gα_q_ and an extracellular uncharged site on the mAChR (Hollmann et al., 2001). In any case, our functional assessment clearly shows that both mI_CAT_ and tonic component of carbachol-induced ileal contraction, in the maintenance of which mI_CAT_ is supposed to play a major role (as the major depolarizing current for the activation of L-type Ca^2+^ channels), are inhibited by ketamine with very similar kinetics (compare Figures 4A,B and Figure 6A).

The evidence that TRPC4 channels are not directly affected by ketamine can be summarised as following: 1) current inhibition developed rather slowly, with time constant of about 1 min (Figure 3A and Figures 4A,B), 2) it was associated with altered voltage-dependence and deactivation kinetics of mI_CAT_ indicating that ketamine acted as a gating modifier rather than a direct channel pore blocker (Figure 3C–E), and 3) most importantly, EA-induced TRPC4 current was insensitive to ketamine (Figure 5A,B). EA, a sesquiterpene from the bark of *Phyllanthus englerin*, is a novel selective and potent agonist of TRPC4 and TRPC5 channels. It activates TRPC4 channels directly, not via G-proteins pathway, and at the external site of the plasma membrane (Akbulut et al., 2015; Minard et al., 2018).

Nanomollar EA fully recovered CCh-induced mI_CAT_ suppressed by ketamine (Figure 5C). These findings not only contribute to our better understanding of the limited usefulness of anticholinesterases (drugs that reduce breakdown of the physiological neurotransmitter acetylcholine) for the treatment of POI (Lubawski and Saclarides, 2008), but also show that the activity of the muscarinic cation channels initiating cholinergic excitation-contraction coupling in the gut could be restored by channel agonists that do not require activation of mAChRs and G-proteins.

Our study has some limitations. First, quantification of concentration-response dependence proved to be technically difficult since the inhibitory effect of ketamine developed very slowly and it was practically irreversible requiring numerous measurements on separate cells. Second, we cannot exclude some effects of ketamine on TRPC6 channels, which make a minor (≈15%) contribution to mI_CAT_ (Tsvilovskyy et al., 2009). Third, inhibition of L-type Ca^2+^-channels and/or PLC by ketamine is likely to be partly responsible for its effects on SM contractions in our functional tests (Figure 6) as InsP_3_ production and Ca^2+^ influx secondary to mI_CAT_-induced depolarisation are ultimately required for the maintenance of these responses. Clearly, these additional targets of ketamine need to be addressed in future studies.

In summary, we demonstrate for the first time that ketamine suppresses mI_CAT_ carried mainly by TRPC4 channels, an effect that could contribute to the development of POI, while simultaneously propose a novel treatment strategy involving direct activators of these channels.

## Data Availability Statement

The raw data supporting the conclusions of this article will be made available by the authors, without undue reservation.

## Ethics Statement

The animal study was reviewed and approved by Biomedical Ethics Committee of the Bogomoletz Institute of Physiology, National Academy of Science of Ukraine.

## Author Contributions

AZ supervised the project; AZ, MM, and DD designed the study; MM, DD, and LA performed the experiments; MM, DD, and AZ analysed and plotted the data; DD initiated the study and discussed its clinical significance/implications; MM, DD, and LA drafted the manuscript; all authors finalized and approved the final version of the manuscript.

## Funding

This research was supported by the Ministry of Education and Science of Ukraine (grant No. 19BF036-01). The costs of open access publishing were covered by the European Union's H2020 grant agreement “NEUROTWIN” (grant No. 857562).

## Conflict of Interest

The authors declare that the research was conducted in the absence of any commercial or financial relationships that could be construed as a potential conflict of interest.

## Abbreviations

CCh, carbachol; EA, (-)-englerin A; GI, gastrointestinal; Gmax, maximal conductance; I-V, current-voltage; mAChR, muscarinic acetylcholine receptor; mI_CAT_, muscarinic receptor cation current; POI, postoperative ileus; SM, smooth muscle; TRPC4 (or TRPC5, TRPC6), Transient Receptor Potential Canonical subfamily member 4 (or 5, 6); V_1/2_, potential of half-maximal activation.
